# Coupled nitrification and N_2_ gas production as a cryptic process in oxic riverbeds

**DOI:** 10.1038/s41467-021-21400-3

**Published:** 2021-02-22

**Authors:** Liao Ouyang, Bo Thamdrup, Mark Trimmer

**Affiliations:** 1 College of Physics and Optoelectronic Engineering, Shenzhen University, Shenzhen, 518060 China; 2 School of Biological and Chemical Sciences, Queen Mary University of London, London, E1 4NS UK; 3 Nordcee, Department of Biology, University of Southern Denmark, 5230 Odense M, Denmark

**Keywords:** Microbiology, Biogeochemistry, Environmental sciences

## Abstract

The coupling between nitrification and N_2_ gas production to recycle ammonia back to the atmosphere is a key step in the nitrogen cycle that has been researched widely. An assumption for such research is that the products of nitrification (nitrite or nitrate) mix freely in the environment before reduction to N_2_ gas. Here we show, in oxic riverbeds, that the pattern of N_2_ gas production from ammonia deviates by ~3- to 16-fold from that predicted for denitrification or anammox involving nitrite or nitrate as free porewater intermediates. Rather, the patterns match that for a coupling through a cryptic pool, isolated from the porewater. A cryptic pool challenges our understanding of a key step in the nitrogen cycle and masks our ability to distinguish between sources of N_2_ gas that 20 years’ research has sought to identify. Our reasoning suggests a new pathway or a new type of coupling between known pathways in the nitrogen cycle.

## Abstract (web summary)

The N cycle involves complex, microbially-mediated shuttling between ammonium, nitrite and nitrate, with climatically important greenhouse gas byproducts. Here the authors use isotope labeling experiments in river sediments and find a cryptic new step in the N cycle between nitrification and the removal of fixed N through N_2_ gas production.

## Introduction

Nitrogen is a key bio-element for life on Earth, integral to proteins and the very DNA that tells life what to do. A vast reservoir of nitrogen resides in the atmosphere as N_2_ gas, unavailable to the majority of life until being fixed by either biological or anthropogenic nitrogen fixation. Life’s organically-bound nitrogen in turn decays to ammonia following excretion or death. To complete the cycle, first nitrogen must be oxidised to nitrite or nitrate which can then be reduced back to atmospheric N_2_ gas. This process of ammonia oxidation—known as nitrification—typically occurs in two stages carried out by specialised aerobic chemoautotrophic ammonia- and nitrite-oxidising microbes, for example, in soils, sediments, freshwater, or marine ecosystems (Eqs. 1 and 2, respectively):

1 $$2{\mathrm{NH}}_4^ + + 3{\mathrm{O}}_2 \to 2{\mathrm{NO}}_2^ - + 2{\mathrm{H}}_2{\mathrm{O}} + 4{\mathrm{H}}^ + \quad\quad{\Delta} G^{\circ \prime} = - 270\;{\mathrm{kJ}}\;\left( {{\mathrm{per}}\;{\mathrm{NH}}_4^ + } \right)$$

2 $$2{\mathrm{NO}}_2^ - + {\mathrm{O}}_2 \to 2{\mathrm{NO}}_3^ - \quad\quad\quad\quad\quad\quad\quad\quad \Delta G^{\circ \prime} = - 79\;{\mathrm{kJ}}\;({\mathrm{per}}\;{\mathrm{NO}}_2^- )$$

Nitrite and nitrate can then be reduced to N_2_ gas either alone, in a phylogenetically widespread form of microbial anaerobic respiration termed denitrification^1^ (Eq. 3a, b) or, in combination with ammonia, in a phylogenetically narrow respiratory pathway termed anaerobic ammonia oxidation, namely anammox^2^ (Eq. 4).

3a $$2{\mathrm{NO}}_3^ - + 10{\mathrm{e}}^ - + 12{\mathrm{H}}^ + \to {\mathrm{N}}_2 + 6{\mathrm{H}}_2{\mathrm{O}}\quad\quad \Delta G^{\circ \prime} = - 360\;{\mathrm{kJ}}\;({\mathrm{per}}\;{\mathrm{NO}}_3^ - )$$

3b $$2{\mathrm{NO}}_2^ - + 6{\mathrm{e}}^ - + 8{\mathrm{H}}^ + \to {\mathrm{N}}_2 + 4{\mathrm{H}}_2{\mathrm{O}}\quad\quad\quad \Delta G^{\circ \prime} = - 282\;{\mathrm{kJ}}\;({\mathrm{per}}\;{\mathrm{NO}}_2^ - )$$

4 $${\mathrm{NH}}_4^ + + {\mathrm{NO}}_2^ - \to {\mathrm{N}}_2 + 2{\mathrm{H}}_2{\mathrm{O}}\quad\quad\quad\quad\quad \Delta G^{\circ \prime} = - 358\;{\mathrm{kJ}}$$

In addition, smaller amounts of N can be returned to the atmosphere as nitrous oxide (N_2_O) but we do not consider those further here^3–5^. Combinations of Eqs. (1) to (4) recycle ammonia back into atmospheric N_2_ gas and this coupling between aerobic nitrification and anaerobic N_2_ gas production is a key concept in the nitrogen cycle, controlling ecosystem production and the abundance of life on Earth^6,7^.

Besides the now accepted reactions described in Eqs. (1) to (4), Broda’s original thermodynamic predictions that drove the quest for anammox^8,9^ also included the potential for complete aerobic ammonia oxidation to N_2_ gas—that, to the best of our knowledge—has yet to be observed in nature:

5 $$4{\mathrm{NH}}_4^ + + 3{\mathrm{O}}_2 \to 2{\mathrm{N}}_2 + 6{\mathrm{H}}_2{\mathrm{O}} + 4{\mathrm{H}}^ + \quad\quad\quad\Delta G^{\circ \prime} = - 316\;{\mathrm{kJ}}\;({\mathrm{per}}\;{\mathrm{NH}}_4^ + )$$

In estuarine or coastal sea sediments, combinations of recognised aerobic and anaerobic metabolisms (Eqs. 1 to 4) buffer the flux of terrestrial nitrogen out to sea and are considered to be physically divided between the oxic and anoxic sediment layers—albeit by only a few tenths of millimetres^10^. In rivers, nitrite and nitrate borne from aerobic nitrification (Eqs. 1 and 2), in either the surrounding catchment soils or the riverbed itself, can be transported over large distances (1–100 km) before some 47 Tg N per year is removed from the fluvial network as N_2_ gas^11–13^. Regardless of the setting, the important point to appreciate here is that the products of aerobic nitrification (e.g., nitrate and nitrite) are assumed to be free to mix with any existing nitrate and nitrite in the surrounding porewater before they are subsequently metabolised, anaerobically, to N_2_ gas. That is, there is—in effect—only one pool of nitrate and nitrite awaiting reduction to N_2_ gas regardless of their origins. Indeed, this concept of free mixing between substrates lies at the very heart of the common ^15^N isotope pairing techniques used to disentangle and quantify the cycling of nitrogen in sediments that are major sources of N_2_ gas on Earth^11,14,15^.

Most research into the coupling between aerobic nitrification and anaerobic N_2_ gas production in sediments has studied the two separately using either oxic or anoxic incubations, respectively^16^, but now work including oxygen is increasing^17^. Previously we demonstrated^18^ that oxic (~30% to 100% of air-saturation for oxygen) gravel and sandy riverbed sediments harbour a coupling between aerobic nitrification and, seemingly, anaerobic N_2_ gas production with that production being attributed to a combination of denitrification and anammox^18^. We now show that the pattern of N_2_ gas production from ammonia in these oxic riverbeds violates the prevailing concept that coupled nitrification and N_2_ gas production is a two-step process with free nitrite or nitrate as intermediates. Not only does this challenge our understanding of a key coupling in the nitrogen cycle but it also masks our ability to distinguish between denitrification and anammox as sources of N_2_ gas. Indeed, it may actually suggest a new pathway or at least a new type of coupling between known pathways in the nitrogen cycle.

## Results and discussion

### N_2_ gas production is independent from porewater nitrite or nitrate

**Fig. 1:**
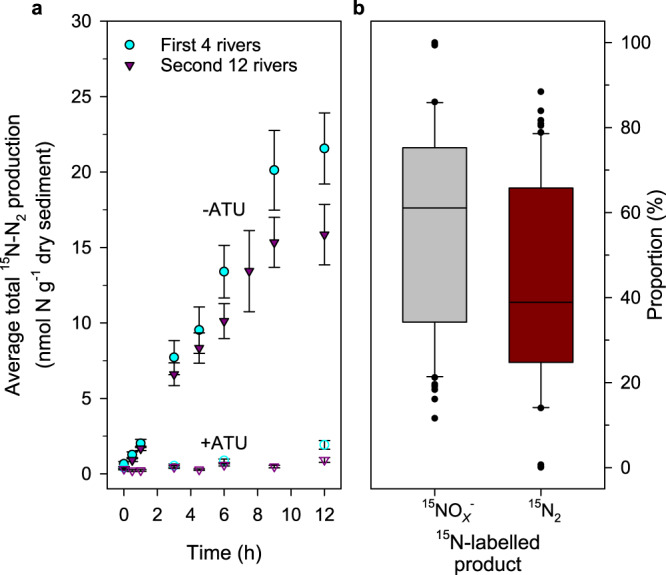
Oxic incubations with ^15^N-ammonia tracer produce both ^15^N_2_ gas and ^15^NO_*x*_^−^. **a** Overall average production of total ^15^N-N_2_ (i.e., ^29^N_2_ and ^30^N_2_) over time in the presence or absence of the inhibitor of ammonia mono-oxygenase, allylthiourea (ATU). The first 4 rivers (cyan circles, *n* = 40, 4 rivers x 5 replicates x 2 treatments at each time point, ± 1 s.e.) and the follow-up across 12 rivers (purple triangles, *n* = 60, 12 rivers x 5 replicates at each time point, ± 1 s.e.); open coloured symbols are the same plus ATU (see Table 1). **b** Proportions of oxidised ^15^N-ammonia tracer from **a**, recovered as either ^15^NO_*x*_^−^ or ^15^N_2_ across the 12 rivers (*n* = 60 as for **a**). Upper and lower box boundaries are 75^th^ and 25^th^ percentiles, respectively, upper and lower whiskers are 90^th^ and 10^th^ percentiles, respectively, the extreme outliers the maxima and minima and the horizontal line the centre, median value.

Following on from our original work^18^ on nitrification and putative anaerobic N_2_ gas production in oxic riverbeds, we wanted to explore further how these two processes are coupled. We began by collecting sediment from four rivers—two each of predominantly gravel and sand and then extended our sampling to a total of twelve rivers (Supplementary Figure 1 and Supplementary Table 1). We added ^15^N-ammonia to oxic sediment microcosms (see Methods) to trace the coupling between nitrification and N_2_ gas production both with and without the inhibitor of aerobic nitrification, allylthiourea^19^ (~80 µM ATU in the porewater, Treatments 1 & 2, Table 1 and Methods) that does not inhibit denitrification or anammox^2,20^. As before^18^, we measured the immediate production of ^15^N-N_2_-gas that was stopped by inhibiting the first step (Eq. 1) of aerobic ^15^N-ammonia oxidation with ATU (Fig. 1a, Table 1). The coupling between aerobic ammonia oxidation and N_2_ gas production was clearly strong, however it was not complete. For example, across the twelve rivers, approximately 60% (Fig. 1b) of the oxidised ^15^N-ammonia tracer was recovered from the porewater as ^15^NO_*x*_^−^, i.e., as either ^15^N-nitrite (Eq. 1) or the final product of nitrification, ^15^N-nitrate (Eq. 2) e.g., ^15^NO_*x*_^−^ is the sum of ^15^NO_2_^−^ and ^15^NO_3_^−^.

**Table 1:** Summary of total ^15^N-N_2_ production in oxic incubations with ^15^NH_4_^+^ or ^15^NO_2_^−^. Mixed-effects models were used to estimate overall rates of total ^15^N-N_2_ production for the incubations in Fig. 1a. Treatments 1 to 6 were applied to sediments from the first set of 4 rivers, and then just treatments 1 and 2 for the subsequent set of 12 rivers. Model fitting was carried out in the lme4 package in R^45^ and rate estimates, standard errors (s.e.) and 95% confidence intervals derived using emtrends from the emmeans package (see Methods). Significant production (**bold**) of ^15^N-N_2_ was only measured with treatments 1 and 3.

Code, Treatment	Rivers (replicates)	Total ^15^N-N_2_ (nmol N g^−1^ h^−1^)	s.e.	Lower 95% C.I.	Upper 95% C.I.
**1**, ^15^NH_4_^+^ + ATU	4 (5)	0.110	0.337	−0.667	0.886
**2**, ^15^NH_4_^+^	4 (5)	**1.855**	0.326	1.078	2.631
**3**, ^15^NH_4_^+^ + ^14^NO_2_^−^ + ATU	4 (5)	0.152	0.337	−0.625	0.929
**4**, ^15^NH_4_^+^ + ^14^NO_2_^−^	4 (5)	**1.941**	0.326	1.165	2.717
**5**, ^14^NH_4_^+^ + ^15^NO_2_^−^ + ATU	4 (5)	0.314	0.326	−0.462	1.091
**6**, ^14^NH_4_^+^ + ^15^NO_2_^−^	4 (5)	0.279	0.326	−0.497	1.055
**1**, ^15^NH_4_^+^ + ATU	12 (5)	0.129	0.178	−0.249	0.506
**2**, ^15^NH_4_^+^	12 (5)	**1.465**	0.176	1.091	1.839

**Fig. 2:**
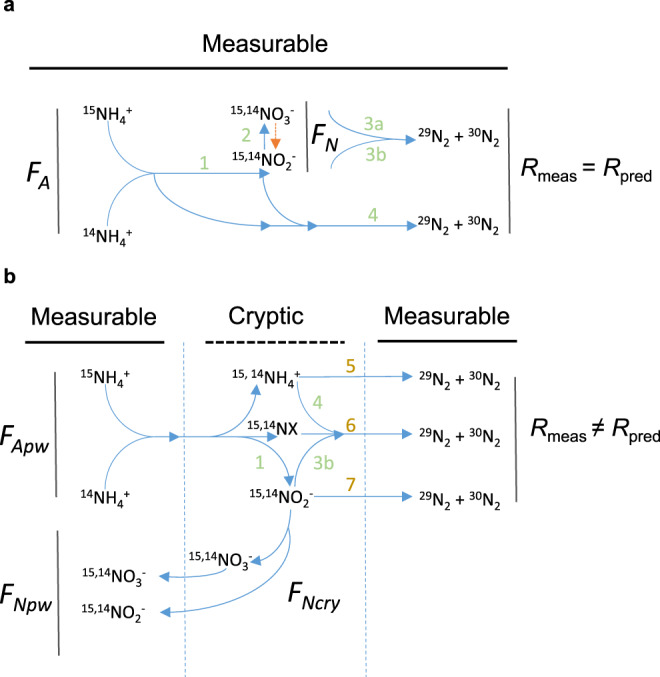
Accepted and proposed cryptic couplings in oxic N cycling. **a** ^15^NH_4_^+^ tracer is added to oxic sediments to mix with ^14^NH_4_^+^ in the porewater, with the fraction of ^15^N labelling known as *F*_*A*_. Through reactions 1 and 2, ^14^NH_4_^+^ and ^15^NH_4_^+^ are oxidised aerobically to ^14,15^NO_2_^−^ and ^14,15^NO_3_^−^ to generate a ^14,15^NO_*x*_^−^ pool with ^15^N labelling known as *F*_*N*_. NO_3_^−^ and/or NO_2_^−^ can be denitrified to N_2_ gas (reactions 3a, 3b), or NO_2_^−^ can oxidise NH_4_^+^ anaerobically through anammox to N_2_ gas (reaction 4). Regardless of the precise setting and combination of reactions, all substrates and products are free to mix and the measured ratio of ^29^N_2_ to ^30^N_2_ produced (*R*) can be predicted from the measured ^15^N labelling in the porewater. The downwards pointing orange arrow indicates NO_3_^−^ respiration to NO_2_^−^ that we do not consider further here. **b** In contrast, our measured values for *R* cannot be predicted using the measured fraction of ^15^N labelling in the porewater (*F*_*A*_ and *F*_*N*_) and known combinations of reactions 1 to 4 but can only be approximated assuming a cryptic element (*F*_*N*cry_). A cryptic element could be a hidden substrate pool (6, novel or known) or novel parts of existing processes (7, e.g., complete nitrifier-denitrification beyond N_2_O to N_2_) and/or a completely new pathway (reaction 5 e.g., complete aerobic ammonia oxidation to N_2_) or cryptic combinations of known pathways after partial aerobic ammonia oxidation to nitrite (reactions, 1, 3b, 4).

The presence of ^15^N-ammonia and ^15^N-NO_*x*_^−^ together in the porewater generates two ^15^N-labelled substrate pools. The fraction of the pool labelled with ^15^N is termed *F*_*A*_ for ammonia (NH_3_) and *F*_*N*_ for NO_*x*_^−^ (Eqs. 10 and 11 in Methods). Theoretically, combinations of Eqs. (1) to (4) can draw on these two substrate pools (*F*_*A*_ and *F*_*N*_) to produce both the single-^15^N-labelled, ^29^N_2_ gas (e.g., ^14^N, ^15^N) and the double-^15^N-labelled, ^30^N_2_ gas (e.g., ^15^N, ^15^N) which we illustrate schematically in Fig. 2a. Note that denitrification can draw on NO_*x*_^−^ as either NO_2_^−^ or NO_3_^−^ but anammox is solely fuelled by NO_2_^−^. The published and accepted mathematical framework^21^ (See derivation of equations in Supplementary Note 1) tells us that the fraction of ^15^N-labelling in each of the substrate pools (*F*_*A*_ and *F*_*N*_) must influence the ratio of ^29^N_2_ to ^30^N_2_ (here termed *R*) and the overall fraction of ^15^N in the N_2_ gas produced e.g., the overall blend of ^28^N_2_, ^29^N_2_ and ^30^N_2_ (here termed *F*_*N*2_)^21,22^. While complex, the accepted framework also tells us that so long as we know what fraction of each component part (*F*_*A*_, *F*_*N*_ and *F*_*N*2_) is labelled with ^15^N, then we can still calculate how the N_2_ gas is produced e.g., by anammox or denitrification and understand the nature of this key coupling in the nitrogen cycle^21,22^.

**Fig. 3:**
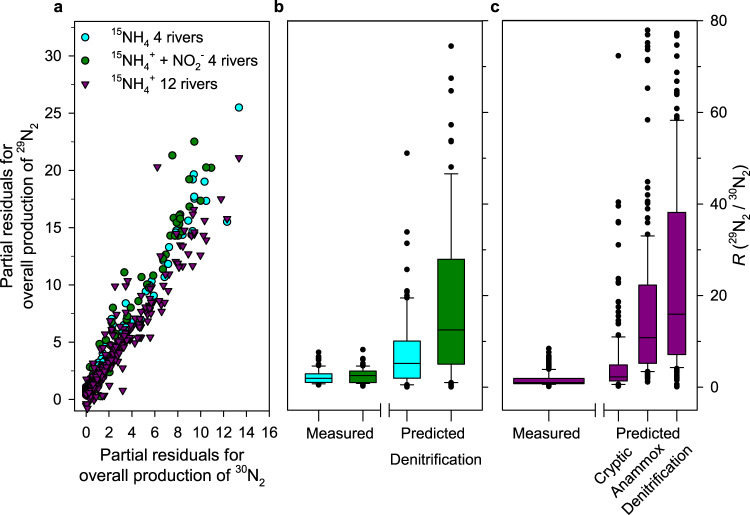
Ratios of ^29^N_2_ and ^30^N_2_ production consistently below those predicted. **a** Consistent ^29^N_2_ production (nmol g^−1^ h^−1^) from ^15^N-ammonia added to oxic sediments, against each corresponding measure of ^30^N_2_ production at each time-point (>0.5 h < 10 h) in each incubation in Fig. 1a presented here as the partial residuals from mixed-effects models (*n* = 100 and *n* = 300, for the 4- and 12-river datasets, respectively). **b** The corresponding measured values for *R* from **a**, for the first 4 rivers incubated with either ^15^NH_4_^+^ (95% CI for *R* = 2.01 to 2.64) or ^15^NH_4_^+^ and additional ^14^NO_2_^−^ (95% CI for *R* = 2.12 to 2.74), against those predicted for denitrification of porewater NO_2_^−^. **c** Measured *R* values for the 12 river sediments incubated with only ^15^NH_4_^+^ (95% CI for *R* = 1.41 to 2.20), against predicted *R* values for denitrification, anammox, and a cryptic coupling. See main text and Table 2. Upper and lower box boundaries are 75th and 25th percentiles, respectively, upper and lower whiskers are 90th and 10th percentiles, respectively, the extreme outliers the maxima and minima and the horizontal line the centre, median value.

We tested the validity of this accepted mathematical framework by changing the fraction of porewater NO_*x*_^−^ labelled with ^15^N (*F*_*N*_) and looking for how this influenced the ratio of ^29^N_2_ to ^30^N_2_ produced (*R*). First we directly decreased *F*_*N*_ by adding ^14^N-nitrite to dilute the ^15^N-nitrite accumulating in the porewater from the oxidation of ^15^N-ammonia (Treatments 3 and 4, Table 1). Surprisingly, diluting *F*_*N*_ had no discernible effect on the values for *R* produced in the two sets of incubations (Fig. 3b. 2.32, 95% CI 2.01 to 2.64 versus 2.43, 95% CI 2.12 to 2.74, see Table 2 and Supplementary Table 2 for ^29^N_2_ and ^30^N_2_ production). We then repeated our incubations with just ^15^NH_4_^+^ (with and without ATU, Treatments 1 and 2) across twelve rivers and measured a similar value for *R* of 1.8 (95% CI, 1.41 to 2.20, Fig. 3c) at an even lower value for *F*_*N*_ (see Table 1). Note, we might have expected *R* to increase steeply as an inverse function of *F*_*N*_ (Supplementary Figure 3). We can predict what values for *R* we might have expected if our N_2_ gas had been produced by either denitrification or anammox fuelled by porewater nitrite and/or ammonia, respectively (Fig. 2a) and compare them to our measured *R* values to highlight the disparity between the two (Fig. 3b, c and Table 2):

6 $${\mathrm{Predicted}}\;{R}\;{\mathrm{for}}\;{\mathrm{denitrification}},\quad R = \frac{{2 \times F_N \times \left( {1 - F_N} \right)}}{{F_N^2}}$$

7 $${\mathrm{Predicted}}\;{R}\;{\mathrm{for}}\;{\mathrm{anammox}},\quad R = \left( {\frac{1}{{F_N}} - 1} \right) + \left( {\frac{1}{{F_A}} - 1} \right)$$

Our measured *R* values were too low to be explained by either denitrification or anammox fuelled by porewater *F*_*N*_ and/or *F*_*A*_ (Fig. 2a) and even a mixture of these two processes couldn’t produce such low values for *R* on average. This consistent disparity between our measured and predicted values for *R*, according to the accepted model, along with the constancy in *R*, despite differences in *F*_*N*_ (Table 2), strongly implies that porewater NO_*x*_^−^ had little influence on the ^15^N-labelling of the N_2_ gas produced from the oxidation of ^15^N-ammonia. Further, in an analogous set of incubations where we added ^15^N-nitrite instead of ^15^N-ammonia, we measured no consistent production of ^15^N-N_2_ gas (Treatments 5 & 6 Table 1 and Methods). Hence, nitrogen for N_2_ formation was not drawn primarily from the porewater NO_*x*_^−^ pool (Fig. 2a). Instead, we propose that any N_2_ producing pathways draw from a cryptic nitrogen pool (Fig. 2b) with ^15^N-labelled fraction, *F*_*N*cry_, instead of the familiar porewater pool with ^15^N-labelled fraction, *F*_*N*pw_. Indeed, if we invoke a cryptic pool by making the ^15^N-labelling of *F*_*N*_ the same as ^15^N-ammonia in the porewater *F*_*A*_ in Eqs. (6) and (7) and thereby force denitrification and/or anammox to draw on that *F*_*N*cry_ pool, then the predicted *R* values come closer to our measured *R* values (*R* cryptic, Fig. 3c and Table 2).

**Table 2:** Summary of the overall measured and predicted ratios of ^29^N_2_ to ^30^N_2_ production (*R*) for treatments 2 and 4 and the fraction of ^15^N labelling in each substrate pool for *F*_*N*_ and *F*_*A*_, on average. Overall measured and predicted *R* estimates, standard errors (s.e.) and 95% confidence intervals were derived with mixed-effects models using lme4 and emmeans (See Methods) and similarly for *F*_*N*_ and *F*_*A*_. See Supplementary Table 3 for further details.

Code, Treatment	Rivers (replicates)	*R* (^29^N_2_/^30^N_2_)	Lower 95% C.I.	Upper 95% C.I.	*P*	*F*_*N*_^‡^		*F*_*A*_^‡^
		*Measured*				NO_2_^−^	NO_*x*_^−^	
**2**, ^15^NH_4_^+^	4 (5)	2.32 (0.16)	2.0	2.6		0.32	0.41	0.57
**4**, ^15^NH_4_^+^ + ^14^NO_2_^−^	4 (5)	2.43 (0.16)	2.1	2.7		0.27	0.36	0.51
**4 minus 2**	4 (5)	0.11 (0.08)			0.20			
**2** & **4**	4 (10)	2.38 (0.15)	2.1	2.7				
**2**, ^15^NH_4_^+^	12 (5)	1.81 (0.20)	1.4	2.2		0.16	0.25	0.45
		*Predicted*^†^						
**2**, ^15^NH_4_^+^	4 (5)	7.81 (1.36)*D*	5.1	10.5				
**4**, ^15^NH_4_^+^ + ^14^NO_2_^−^	4 (5)	20.05 (1.49)*D*	16.9	22.3				
**2**, ^15^NH_4_^+^	12 (5)	29.4 (2.28)*D*	24.8	33.9				
**2**, ^15^NH_4_^+^	12 (5)	19.3 (2.28)*A*	14.8	23.9				
**2**, ^15^NH_4_^+^	12 (5)	9.3 (2.28)*C*	4.8	13.8				

^†^Predicted *R* values, using Eqs. (6) and (7), for denitrification (*D*), anammox (*A*) and cryptic (*C*) processes fuelled by porewater NO_2_^−^, see Supplementary Table 3 for NO_*x*_^−^. Note also that the predicted values are derived using each individual measure of *F*_*N*_ and *F*_*A*_ in each vial, and that *F*_*N*_^‡^ are the overall mean values simply to illustrate the effect of adding ^14^NO_2_^−^ to the incubations with sediments from the first 4 rivers and overall lower *F*_*N*_ value for the 12 river incubations.

### N_2_ is produced from ammonia through a cryptic intermediate

We can use both the accepted^21^ and a new mathematical framework to more formally justify our proposal for a cryptic intermediate pool or process. First, we define the proportion of N_2_ gas coming from anammox relative to denitrification that is conventionally known as *ra*^15^. *ra* has to lie between 0 and 1 and, in the accepted framework, is expressed as a function of porewater *F*_*A*_ and *F*_*N*_ and *R* according to^21^ (See Eq. (1) to (14) in Supplementary Note 1):

8 $$ra = \frac{{(R + 2) \times F_N^2-2 \times F_N}}{{(F_N-F_A) \times [(R + 2) \times F_N-1]}}$$

In the accepted framework, however, our measured values for *R* and porewater *F*_*A*_ and *F*_*N*_ generate nonsensical estimates for *ra* (e.g., −6.06 to 3.03, not > 0 < 1). Just as for Fig. 3c, we cannot apportion N_2_ gas between anammox and denitrification drawing on porewater *F*_*N*_ and/or *F*_*A*_ – in the conventional sense – to produce our measured *R* values (Fig. 2a). Next, we define the ^15^N- labelling of the N_2_ gas produced (*F*_*N2*_), which, like *ra* (Eq. 6), also has to lie between 0 and 1 (See Eq. (1) to (14) in Supplementary Note 1).

9 $$F_{N2} = F_N - \frac{{R \times F_N + 2 \times (F_N-1)}}{{2 \times (R + 2 - \frac{1}{{F_N}})}}$$

Unlike *ra*, which is expressed as a function of both porewater *F*_*A*_ and *F*_*N*_, only *F*_*N*_ is required to parameterise *F*_*N2*_ (Eq. 9*cf*. Eq. 8). That is not to say that *F*_*A*_ has no influence on *F*_*N2*_, as *F*_*N*_—be it either the *F*_*N*cry_ or *F*_*N*pw_ pools—must result from ammonia oxidation drawing on *F*_*A*_ (Fig. 2).

**Fig. 4:**
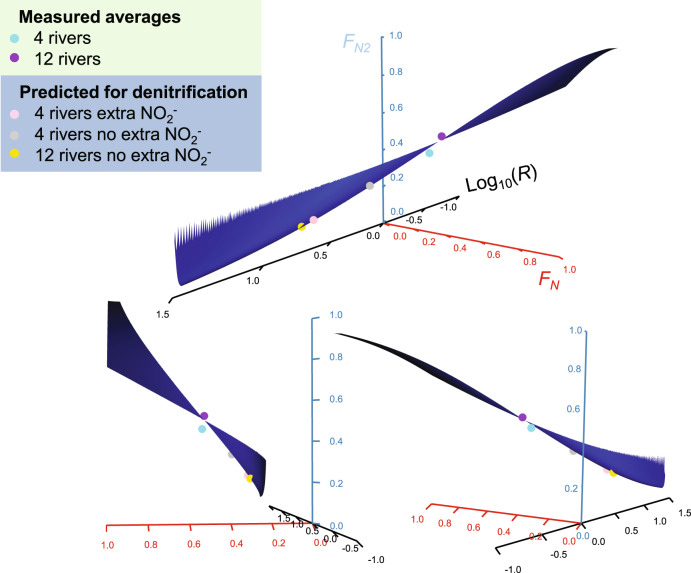
Orientations of the solution space ribbon with both measured and predicted values for *R*. Here we present all data in just one solution space for the average fraction of ^15^N in the ammonia pool (*F*_*A*_) of 0.51 and combinations of Eq. (8) (*F*_*N*2_) and 9 (*ra*) both yielding values between > 0 < 1. *R* is the ratio of ^29^N_2_ to ^30^N_2_ and *F*_*N*_ and *F*_*N*2_ the fraction of ^15^N in the NO_*x*_^−^ and N_2_ gas pools, respectively. To plot *F*_*N*2_ for each of our measured values of *R* we have to assume that *F*_*N*_ equals *F*_*A*_ measured in the porewater. In the solution space, there are no solutions where *F*_*A*_ = *F*_*N*_ (i.e., 0.51) and this singularity appears as a gap in the ribbon. Despite measurable changes in porewater *F*_*N*_, the average values for both the 4-river and 12-river study appear near to each other and the gap where *F*_*A*_ = *F*_*N*_. Note that the better parameterised 12-river average touches the gap and by inference, *F*_*A*_ ≈ *F*_*N*cry_ (Fig. 2b). Denitrification fuelled by porewater NO_*x*_^−^ predicts values away from our measured values for *R*. Note, for the single predicted denitrification *R* values we use the median *F*_*N*_ values.

We can then use solutions to Eqs. (8) and (9) between > 0 < 1 to define a solution space for any combination of *F*_*N*_, *F*_*A*_*,* and realistic values for *R* (See Supplementary Figure 3 for *R* as a function of ^15^N atom %) that we can visualise as a 3D ribbon (Fig. 4). The height of the ribbon is defined in terms of *F*_*N2*_ and is depicted here for our average value for *F*_*A*_ of 0.51 (Table 1 and see Supplementary Fig. 4 for *F*_*A*_ at 0.1 and 0.9). Overall the ribbon is very narrow and where *F*_*A*_ = *F*_*N*_ there are no solutions and this singularity appears as a gap in the ribbon. If *F*_*N*cry_ is isolated and derives solely from the oxidation of *F*_*A*_ (Fig. 2b), then *F*_*N*cry_ has to equal *F*_*A*_. Further, if *F*_*N*2_ is only dependent on *F*_*N*_ (Eq. 9) and this *F*_*N*_ is equivalent to *F*_*N*cry_, then our calculated values for *F*_*N2*_—plotted as functions of our measured values for *R* and *F*_*A*_ (where *F*_*N*cry_ equal *F*_*A*_)—should fall near the gap in the ribbon where *F*_*N*_ equals *F*_*A*_. This is indeed what we observe and especially for the better parameterised 12 river estimate (Fig. 4). In contrast, if we again force denitrification to be the only source of N_2_, and calculate *F*_*N2*_ assuming that *F*_*N*_ = *F*_*N*pw_ (Fig. 2a), then the points fall away from our measured *R* values. Hence, in the presence of ^15^N-ammonia and oxygen, our measured *R* values only make sense if we assume *F*_*N*cry_ = *F*_*A*_ (Fig. 2b) i.e., the porewater nitrite pool essentially represents the left-overs of the cryptic transformations during which N_2_ is produced.

### Internal NO_*x*_^−^ cycling or a novel pathway or organism

We propose that the coupling between ammonia oxidation and N_2_ gas production in oxic, permeable riverbed sediments involves a cryptic intermediate pool derived solely from the oxidation of ammonia that remains isolated from the porewater prior to the production of N_2_ gas. In one scenario, a cryptic pool, similar to the porewater NO_*x*_^−^ pool, is fed by the oxidation of ammonia to NO_*x*_^−^, or possibly NO (ref. ^3,23,24^), through nitrification. The pathway from *F*_*N*cry_ to the production of N_2_ gas, however, branches off before that NO_*x*_^−^ mixes with the ambient porewater NO_*x*_^−^ (Fig. 2b) and would require internal NO_*x*_^−^ cycling. Internal NO_*x*_^−^ cycling is recognised as a potential source of interference for ^15^N isotope tracer studies in the ocean^25,26^ and is known in the consortia of ammonia oxidisers and anammox bacteria in wastewater CANON^27^ reactors (Complete Autotrophic Nitrogen removal Over Nitrite. Figure 2b, reactions 1 & 4) – though the actual mechanism in nature remains unknown.

Alternatively, some aerobic ammonia oxidising bacteria first produce nitrite (reaction 1) that they then reduce to N_2_O gas in a process known as nitrifier-denitrification^3^. Known nitrifier-denitrifier bacteria, however, lack a canonical N_2_O-reductase (NOS, *nosZ*) to reduce N_2_O to N_2_ gas, so are not currently recognised as complete denitrifiers (reaction 7, Fig. 2b). Nitrosocyanin, a soluble red Cu protein isolated from *Nitrosomonas europaea*^28^, is recognised as a plausible substitute to canonical N_2_O-reductase that could enable complete nitrifier-denitrification to N_2_ gas^3^. Our data enable us to test this hypothesis. For example, we know that ^15^NO_2_^−^ from the initial oxidation of ^15^NH_4_^+^ exchanges with the porewater (reaction 1, Figs. 1b and 2a) and we would expect, therefore, that ^15^NO_2_^−^ added to the porewater would be available to any nitrifying-denitrifying bacteria^29^. We have, however, already shown that adding ^15^NO_2_^−^ to the porewater resulted in no consistent production of N_2_ gas (Treatments 5 & 6, Table 1) i.e., N_2_ gas production is dependent on the initial oxidation of ^15^N-ammonia. This fact, along with the clear discrepancy between the measured and predicted scenarios involving porewater NO_*x*_^−^ (Figs. 3b, 3c & 4) make it hard to reconcile our N_2_ gas production with either nitrifier-denitrification or canonical denitrification (reactions 3a, 3b & 7, Fig. 2).

Finally, it is theoretically possible for ammonia to be completely oxidised by oxygen to N_2_ gas (equation 5^8^) within a single, unknown organism. Such a reaction offers the simplest explanation for our results, with their strong dependency on aerobic ammonia oxidation and lack of influence from external porewater nitrite. Regardless of the actual pathway that produces the N_2_ gas (Fig. 2b), an isolated cryptic intermediate pool has to have the same ^15^N-labelling of the ammonia pool (*F*_*N*cry_ = *F*_*A*_). As a consequence of this equality, we can no longer distinguish between sources of N_2_ gas, be it a denitrification-like pathway reductively combining N from an oxidised cryptic pool, an anammox-like process drawing on ammonia and cryptic N, or complete ammonia oxidation, as they would all produce ^29^N_2_ and ^30^N_2_ at the same ratio (Fig. 2b where *R* is equal for each process).

Our observations challenge the current understanding of a key coupling in the nitrogen cycle in permeable, oxic riverbed sediments that may also apply to other biomes where the oxidation of ammonia is tightly coupled to the production of N_2_ gas, such as continental shelf-sediments^30,31^ and groundwater aquifers^17^. Whether it transpires that our cryptic coupling is mediated by a novel organism or, as of yet, a masked combination of known players in the nitrogen cycle remains to be resolved.

## Methods

### Study sites and sediment sampling

We began by collecting sediment samples from four rivers which we subsequently widened to a total of twelve rivers in southern England, UK, between October 2015 and May 2016 (Supplementary Figure 1 and Supplementary Table 1). Among them, the Rivers Lambourn, Darent, Wylye, Rib, Pant, Stour (1) and Stour (2) have chalk-based, permeable gravel-dominated riverbeds, while the Rivers Marden, Hammer, Medway, Broadstone, and Nadder have less permeable, sand-dominated riverbeds as described elsewhere^18,32,33^. At each river, surface sediments (<5 cm) were collected from five different locations using Perspex corers (13-cm × 9-cm internal diameter, 827 mL and sealed at one end with an oil-seal stopper)) which were then transferred to plastic zip-lock bags (VWR International) and stored in a cool bag (Thermo) during transport back to the laboratory. Each sediment sample from each river was then homogenised in the laboratory for the experiments described below.

#### Aerobic ammonia oxidation in oxic sediment slurries

^15^N-NH_4_^+^ oxidation experiments were carried out with sediments first from four rivers (the rivers Lambourn, Wylye, Marden, and Hammer) and then all twelve. In a standard anoxic application of ^15^N isotope pairing techniques^34–36^, ambient porewater nitrite, nitrate, and any residual oxygen are removed by pre-incubating the anoxic sediment slurries for 12 h to 24 h before adding any ^15^N-tracers^35,36^. Here this was not possible as we were measuring the aerobic oxidation of NH_4_^+^ and so to avoid contamination from the high background ^14^NO_*x*_^−^ (^14^NO_3_^−^ + ^14^NO_2_^−^), which is typical for these rivers^24^, instead we used nitrite- and nitrate-free synthetic river water (0.12 g/l NaHCO_3_, 0.04 g/l KHCO_3_, 0.07 g/l MgSO_4_^.^7H_2_O, 0.09 g/l CaCl_2_ 2H_2_O, pH = 7) to make the sediment slurries as before^18^.

Oxic slurries were prepared by adding approximately 3 g sediment (~0.75 ml of porewater) and 2.7 ml air-saturated synthetic river water into 12 ml gas-tight vials (Exetainer, Labco), leaving an approximate 6 ml headspace of air which is equivalent to ~58 µmol O_2_ per prepared vial. We know from previous incubations with similar sediments from 28 rivers^37^ respiration rates to be ~187 nmol O_2_ g^−1^ h^−1^, on average (±64.3, 95%, C.I.), that would consume ~12% of the total oxygen during a 12 h incubation. In addition, we also checked oxygen over time using a microelectrode (50 µm, Unisense) in parallel sets of scaled-up slurries (120 mL with the same ratio of sediment to water to headspace) for two rivers and found comparatively little consumption as before^18^ and see example in Supplementary Figure 2.

To trace the oxidation of ammonia to N_2_ gas, the prepared oxic slurry vials were then sealed and injected with 100 µl of 14 mM ^15^NH_4_^+^ stock-solutions (98 atom% ^15^N, Sigma-Aldrich) to generate final porewater concentrations of ~390 µM ^15^NH_4_^+^. This high ^15^N concentration ensured sufficient labelling of the ammonia pool (~50%) to enable quantifiable production of both single-labelled, ^29^N_2_, and dual-labelled, ^30^N_2_, in order to calculate *R* in Eqs. (6) to (9). To link the production of N_2_ gas to the initial aerobic oxidation of ammonia, an additional set of slurries were injected with 100 µl of 14 mM ^15^NH_4_^+^ (as above), along with 2.8 mM (stock-solution) of the ammonia mono-oxygenase inhibitor^19^, allylthiourea (ATU), to give final porewater concentrations of ~390 µM ^15^NH_4_^+^ and ~80 µM ATU. While we have shown previously that 80 µM ATU inhibits aerobic ammonia oxidation in gravel and sandy riverbed sediments^18^, higher concentrations maybe required in other settings^38^. All of the oxic slurry vials were then incubated on a shaker (120 rpm, Stuart SSL1) for up to 12 h (Table 1, Treatments 1 and 2) in a temperature-controlled room at 12 °C. Incubations amended with just ^15^NH_4_^+^ were terminated at 0 h, 0.5 h, 1 h, 3 h, 4.5 h, 6 h, 9 h, and 12 h while those amended with both ^15^NH_4_^+^ and ATU were terminated at 0 h, 3 h, 6 h, and 12 h by injecting 100 μl of formaldehyde (38%, w/v) through the vial septa. All vials were then stored upside down prior to quantification of ^29^N_2_ and ^30^N_2_ by mass-spectrometry and *R* is then simply ^29^N_2_/^30^N_2_ (see below).

In addition to measuring the production of ^29^N_2_ and ^30^N_2_ gases (*R*), the fraction of ^15^N in the inorganic nitrogen porewater pools (*F*_*A*_ for ammonia and *F*_*N*_ for NO_*x*_^−^ e.g., NO_2_^−^ plus NO_3_^−^) needed to be quantified too (see Eqs. 6 to 9). To avoid any potential interference from formaldehyde, on the analysis of the inorganic nitrogen species, a parallel set of ^15^NH_4_^+^ amended slurries was prepared solely for nutrient analyses. At each time point (as above for N_2_ gas analysis), vials were injected with 20 µL of 1.6 M NaOH to preserve nitrite before being frozen at −20 °C^39^. Samples were defrosted and centrifuged at 1200 rpm for 10 min and the collected supernatant analysed (see below).

#### Manipulating the degree of ^15^N-labelling in the porewater NO_2_^−^ pool (*F*_*N*_ as *F*_*N*pw_)

In typical anoxic sediment slurry incubations used to quantify N_2_ gas production from denitrification and anammox^34,35^, the fraction of porewater substrate labelled with ^15^N (*F*_*A*_ or *F*_*N*_) influences the ratio of ^29^N_2_ to ^30^N_2_ produced. To characterise the influence of porewater NO_2_^−^ on the coupling between ^15^N-NH_4_^+^ oxidation and ^15^N-N_2_ production in oxic sediment slurries, we manipulated the fraction of porewater NO_2_^−^ labelled with ^15^N. Oxic sediment slurries from the first four riverbeds were injected (100 µl) with combinations of stock-solutions of 14 mM ^15^NH_4_^+^ and 840 µM ^14^NO_2_^−^ or just 14 mM ^15^NH_4_^+^ and both with or without 2.8 mM ATU. This generated final porewater concentrations of ~390 µM ^15^NH_4_^+^, ~24 µM ^14^NO_2_^−^ or ~80 µM ATU and the prepared vials were then incubated on a shaker as above (see Table 1, Treatments 3 and 4). As above, oxic slurry vials were sacrificed at different time points for ^15^N_2_ gas analysis and with a parallel set of ^15^NH_4_^+^ or ^15^NH_4_^+^ plus NO_2_^−^ amended slurries solely for nutrient analyses.

To further test the dependency of N_2_ gas production on the initial oxidation of ^15^N-ammonia, we also performed a set of analogous incubations with sediments from the first four rivers with ^15^NO_2_^−^ (Table 1, Treatments 5 and 6). Here everything was the same (amount of sediment, with or without ATU, incubation times, oxygen etc.,) except the ^15^N-labelling was added with nitrite rather than ammonia (as above) to final concentrations of ~390 µM ^14^NH_4_^+^ and ~24 µM ^15^NO_2_^−^ (98 atom% ^15^N, Sigma-Aldrich). If active, we would have expected N_2_ gas production from reactions 3b and 4.

#### Analytical methods

Headspaces of the oxic slurry samples were analysed for ^15^N-N_2_ using a continuous-flow isotope ratio mass spectrometer (Sercon 20–22, UK) as described elsewhere^18^. The mass spectrometer has a sensitivity of 0.1 ‰ ^15^N which here translates to approximately 0.1 nmol ^15^N-N_2_ g^−1^ dry sediment. To determine porewater *F*_*N*_ (NO_2_^−^ or NO_*x*_^−^, below) the concentration of ^15^NO_2_^−^ in the ^15^NH_4_^+^ treatments was measured, the preserved supernatants were diluted and 3 ml of sample transferred into a new 3 ml gas-tight vial (Exetainer, Labco), the vial capped and a 0.5 ml helium headspace (BOC) added. Samples were injected with 100 μl of sulfamic acid (4 mM in 4 M HCl) and placed on a shaker (120 rpm, Stuart SSL1) overnight to reduce ^15^NO_2_^−^ to ^15^N-N_2_ and the headspaces subsequently analysed for ^15^N-N_2_ as above^18,40^. For ^15^NO_*x*_^−^ (^15^NO_2_^−^ plus ^15^NO_3_^−^) analysis, 0.3 g spongy cadmium and 200 µl of 1 M imidazole, along with 3.5 ml of sample were added to each gas-tight vial (12 ml, Exetainer, Labco) and the vials shaken (120 rpm, Stuart SSL1) for 2.5 h to reduce ^15^NO_3_^−^ to ^15^NO_2_^−^ and the samples then treated as above to convert ^15^NO_2_^−^ to N_2_^18,41^. The sensitivity for ^15^NO_*x*_^−^ was approximately 0.4 nmol ^15^N g^−1^ dry sediment. *F*_*N*_ was then calculated for NO_2_^−^ or NO_*x*_^−^ as:

10 $$F_N = \frac{{{\,}^{15}{\mathrm{NO}}_x^ - }}{{({\,}^{15}{\mathrm{NO}}_x^ - + {\,}^{14}{\mathrm{NO}}_x^ - )}}$$

And similarly for *F*_*A*_:

11 $$F_A = \frac{{{\,}^{15}{\mathrm{NH}}_4^ + }}{{\left( {{\,}^{15}{\mathrm{NH}}_4^ + + {\,}^{14}{\mathrm{NH}}_4^ + } \right)}}$$

Where ^15^NH_4_^+^ was determined by the increase in concentration, measured by standard indophenol-blue wet-chemistry, above ambient background in controls after the addition of ^15^NH_4_^+^.

Sediment particle size was determined by sorting the dried sediments through a series of sieves (Endecotts Ltd, England) from 16 mm, 13.2, 8, 4, 1.4, 0.5, 0.25, 0.125, to 0.0625 mm and then weighing each size fraction. Grain size distributions were calculated and classified on the Wentworth scale as gravel (particles coarser than 2 mm), sand (particles between 0.0625 and 2 mm), mud (silt plus clay material finer than 0.0625 mm)^42^. For sediment organic C and N content, disaggregated samples were oven-dried, acidified by HCl (2 M) to remove inorganic carbonates^43^ and re-dried to a constant weight. Then ~50 mg of sediments were transferred to tin-cups, reweighed, and combusted at 1000 °C in an integrated elemental analyser and mass-spectrometer (Sercon, Integra 2, UK).

#### Statistical analysis

We used mixed-effects models to estimate overall rates of total ^15^N-N_2_ gas production during the incubations (Fig. 1a), treating each of either the first four or subsequent twelve rivers as genuine, independent replicates. Within each river, each of the 5 technical replicates were nested within each respective river and fitted as random effects on the slope and intercept in each case; though it was not always necessary to retain replicate or all the random effects in a model to get the best fit to the data – based on lowest AIC (Akaike Information Criterion). To visualise the consistent production of ^29^N_2_ to ^30^N_2_ across the incubations with ^15^N-ammonia, we regressed each measure of ^29^N_2_ on each measure of ^30^N_2_, at each time point, in each incubation and display (Fig. 3a) the partial residuals for the best fitting model^44^. To estimate the overall average measured and predicted ratios of ^29^N_2_ to ^30^N_2_ (*R*) we only used the data for the time points >0.5 h < 10 h i.e., when there was measurable (~0.1 nmol N_2_ g^−1^ dry sediment), steady-production of both ^15^N labelled gases, divided each measure of ^29^N_2_ by each respective measure of ^30^N_2_ at each time point, in each incubation and treated river and replicate as above. For the first 4 rivers, the ratio *R* was estimated by fitting each time point as a random-effect, but for the larger, 12 river dataset, time was fitted as a fixed-effect and *R* estimated for the middle time point in the incubations and similarly for *F*_*N*_ (for both NO_2_^−^ and NO_*x*_^−^) and *F*_*A*_. All statistical analyses were performed in R (version 3.6.3, 2020-02-29) under RStudio (version 1.2.5033). Model fitting was carried out in the “lme4” package (version 1.1-21) and parameter (marginal mean) estimates, standard errors, and confidence intervals derived using the “emmeans” package (version 1.4.5) with Kenwood-Roger degrees of freedom and Tukey correction where appropriate.

## Supplementary information

Supplementary Information

Peer Review File

Supplementary Data 1

Description of Additional Supplementary Files

## Data Availability

Source data are provided with this paper.

## Source data

Source Data
