# Supplementary material for: Coupled nitrification and N2 gas production as a cryptic process in oxic riverbeds
Source: Nat Commun. 2021 Feb 22;12:1217. doi: 10.1038/s41467-021-21400-3 (PMC7900231; doi:10.1038/s41467-021-21400-3)
Supplement: Supplementary file 1 — Supplementary Information [file 41467_2021_21400_MOESM1_ESM.pdf]

Supplementary Information

Coupled nitrification and N_2 gas production as a cryptic process in oxic riverbeds

Ouyang et al.

Supplementary Note 1 | Derivation of equations. To derive equations 8 and 9 in the main text in order to define the solution space (Figure 4) where both *ra* (by convention the relative contribution from anammox to total N_2 production[1]) and F_{N2} fall between >0<1.

Let *D28*, *D29*, and *D30* represent the rates of $^{28}N_2$, $^{29}N_2$, and $^{30}N_2$ production (here in nmol N g^{-1} dry sediment h^{-1}) by denitrification, *D*, respectively. From denitrification, N_2 is generated through random isotope pairing. Thus, with F_N being the fraction of the total porewater nitrite pool labelled with ^{15}N (or the overall NO_x^- pool, $NO_2^- + NO_3^-$):

$$D30 = D \times F_N^2 \qquad 1$$

$$D29 = D \times 2 \times F_N \times (1 - F_N) \qquad 2$$

$$D28 = D \times (1 - F_N)^2 \qquad 3$$

If denitrification is the only source of N_2, then the fraction of ^{15}N in the N_2 gas produced is the same as in the porewater nitrite source pool and, combining *1* and *2*, is given by:

$$F_{N2\text{-}denitrification} = F_N = \frac{1}{1 + \frac{D29}{2 \times D30}} \qquad 4$$

N_2 from anammox (e.g. nmol N g^{-1} dry sediment h^{-1}) is generated by a 1:1 pairing between N from ammonia and nitrite. Thus, if *A28*, *A29*, and *A30* represent the production of $^{28}N_2$, $^{29}N_2$, and $^{30}N_2$ from anammox, *A*, and with F_A being the fraction of the total porewater ammonia pool labelled with ^{15}N:

$$A30 = A \times F_A \times F_N \qquad 5$$

$$A29 = A \times [F_A \times (1 - F_N) + F_N \times (1 - F_A)] \qquad 6$$

$$A28 = A \times (1 - F_A) \times (1 - F_N) \qquad 7$$

and the degree of ^{15}N labelling in the N_2 produced through anammox is given by:

$$F_{N2\text{-}anammox} = \frac{2 \times A30 + A29}{2 \times (A30 + A29 + A28)} \qquad 8$$

Inserting equations 5 to 7 in 8 and cancelling out A:

$$F_{N2\text{-}anammox} = 0.5 \times (F_A + F_N) \qquad 9$$

Let ra represent the relative contribution of anammox to N_2 production:

$$ra = \frac{A}{A+D} \qquad 10$$

then:

$$F_{N2} = ra \times F_{N2\text{-}anammox} + (1 - ra) \times F_{N2\text{-}denitrification} \qquad 11$$

and inserting 4 and 9 in 11:

$$F_{N2} = ra \times 0.5 \times (F_A + F_N) + (1 - ra) \times F_N = ra \times 0.5 \times (F_A - F_N) + F_N \qquad 12$$

With both F_{N2} and ra unknown, this cannot be solved. Instead, as shown in[2] we can determine ra from the ratio, R, of $^{29}N_2$ and $^{30}N_2$ production:

$$ra = \frac{(R+2) \times F_N^2 - 2 \times F_N}{(F_N - F_A) \times [(R+2) \times F_N - 1]} \qquad 13$$

and inserting in 12 we derive F_{N2} solely from F_N and R:

$$F_{N2} = F_N - \frac{R \times F_N + 2 \times (F_N - 1)}{2 \times (R + 2 - \frac{1}{F_N})} \qquad 14$$

Note that F_A is no longer required.

Supplementary Figure 1. Approximate locations of the 12 rivers in the south east of England, UK. See Supplementary Table 1 for each latitude and longitude and some sediment characteristics at each site.

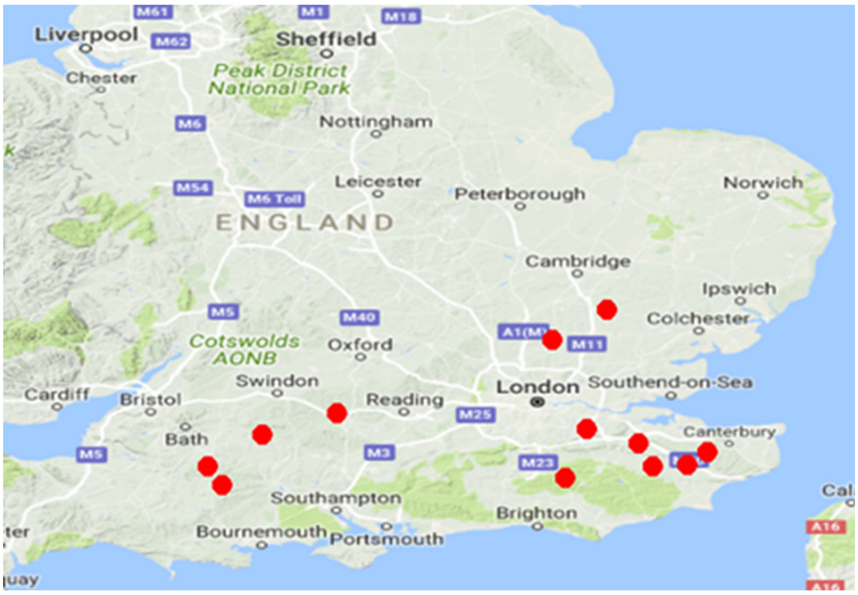

Supplementary Figure 2. Example of an oxic incubation with $^{15}NH_4^+$ both with and without ATU (allylthiourea) for a gravel-dominated sediment from the River Darent. **a,** Production of $^{15}N\text{-}N_2$ (○ –ATU, ▽ + ATU) and dissolved oxygen concentrations throughout the incubation (■) and **b,** parallel production of $^{15}N\text{-}NO_3^-$ (same symbols as **a**). Data are mean values ± standard error (n = 5) and the maintenance of oxic conditions throughout agrees with what we reported previously[3].

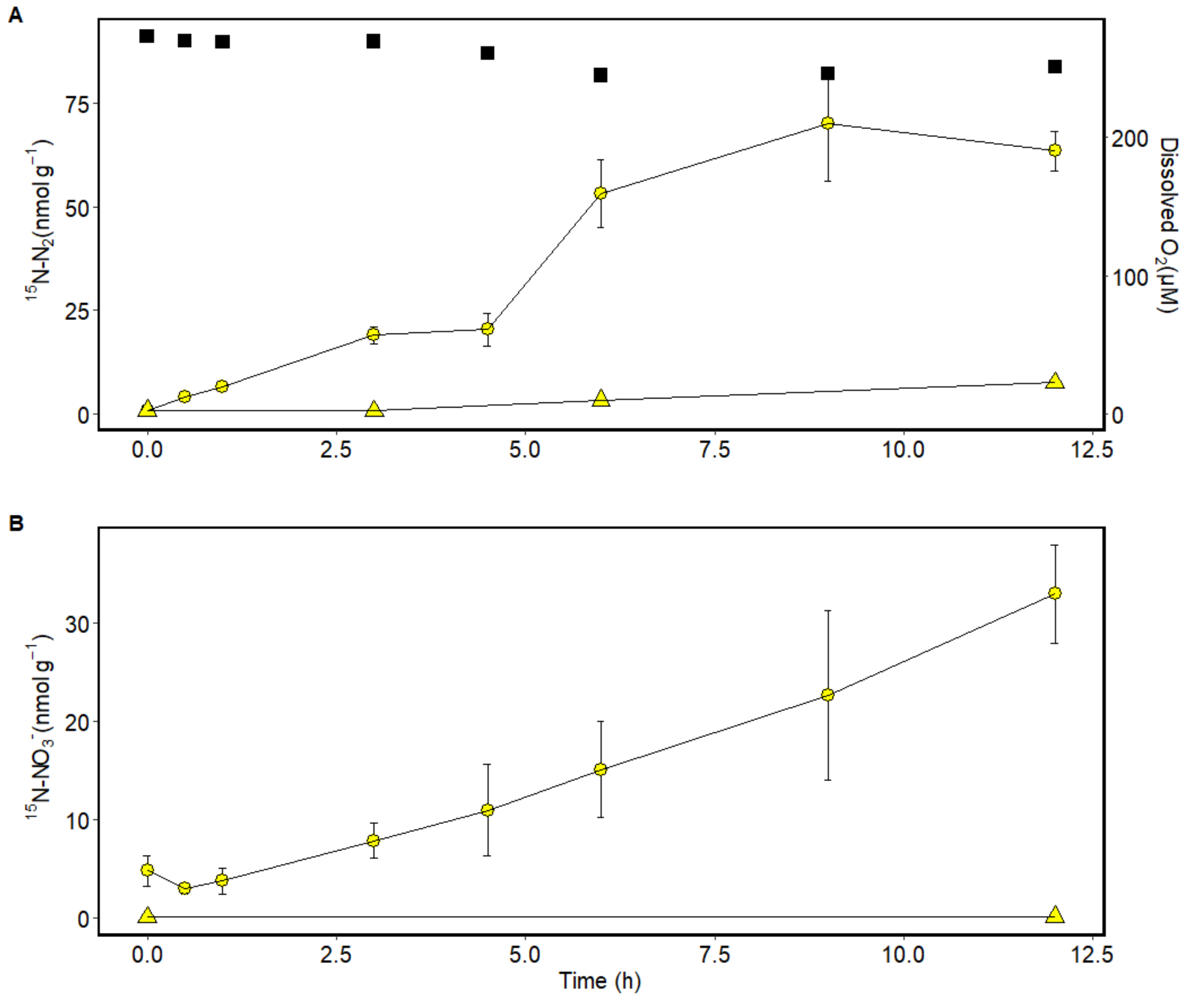

Supplementary Figure 3. In a typical ^{15}N isotope pairing experiment ^{15}N-labelled nitrite or nitrate could be added at purities from 5% to 99.2% (^{15}N atom %) to give a range of F_N where F_N = ^{15}N atom % / 100. If the resulting ^{15}N-labelled N_2 gases were simply produced by denitrification, then the ratio between $^{29}N_2$ and $^{30}N_2$ (R) would increase as an inverse function of ^{15}N (atom %). **a,** Original, linear data and **b,** after $\log_{10}$ transformation as for the *x*-axis in Figure 4 in the main text.

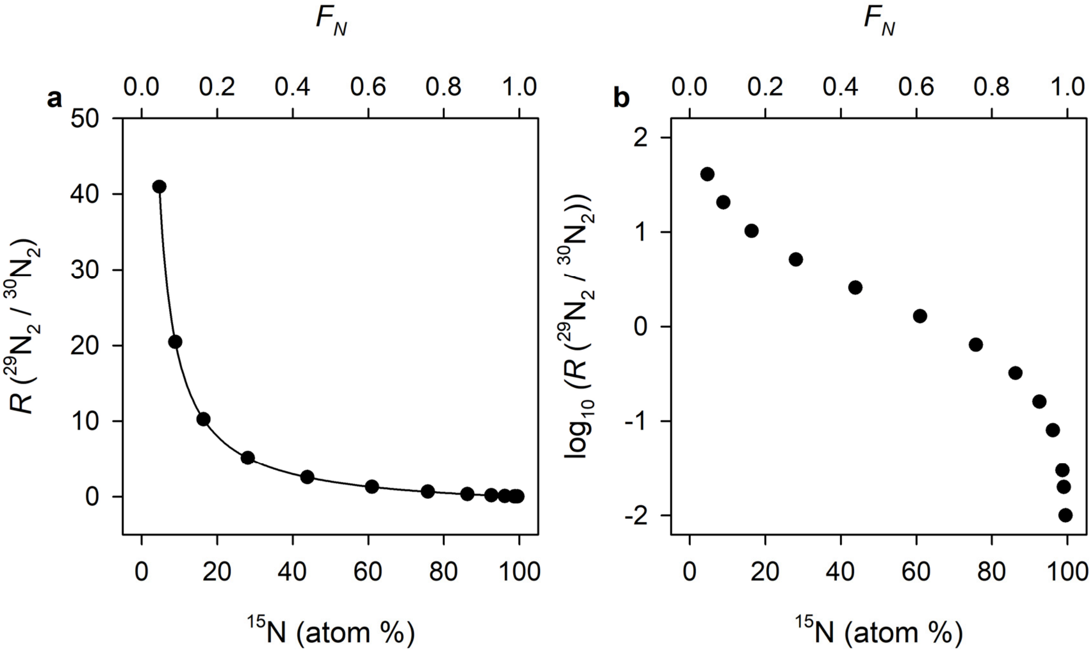

Supplementary Figure 4. Solution spaces for low (0.1) and high (0.9) values for F_A and combinations of equations 8 and 9 where both F_{N2} and *ra* fall between >0<1. Note the shift in the position of the gap, where F_A=F_N, from *R* of between ~ 1.0 and 1.5, to *R* of between ~ -0.6 to -0.7 for the low and high values of F_A, respectively.

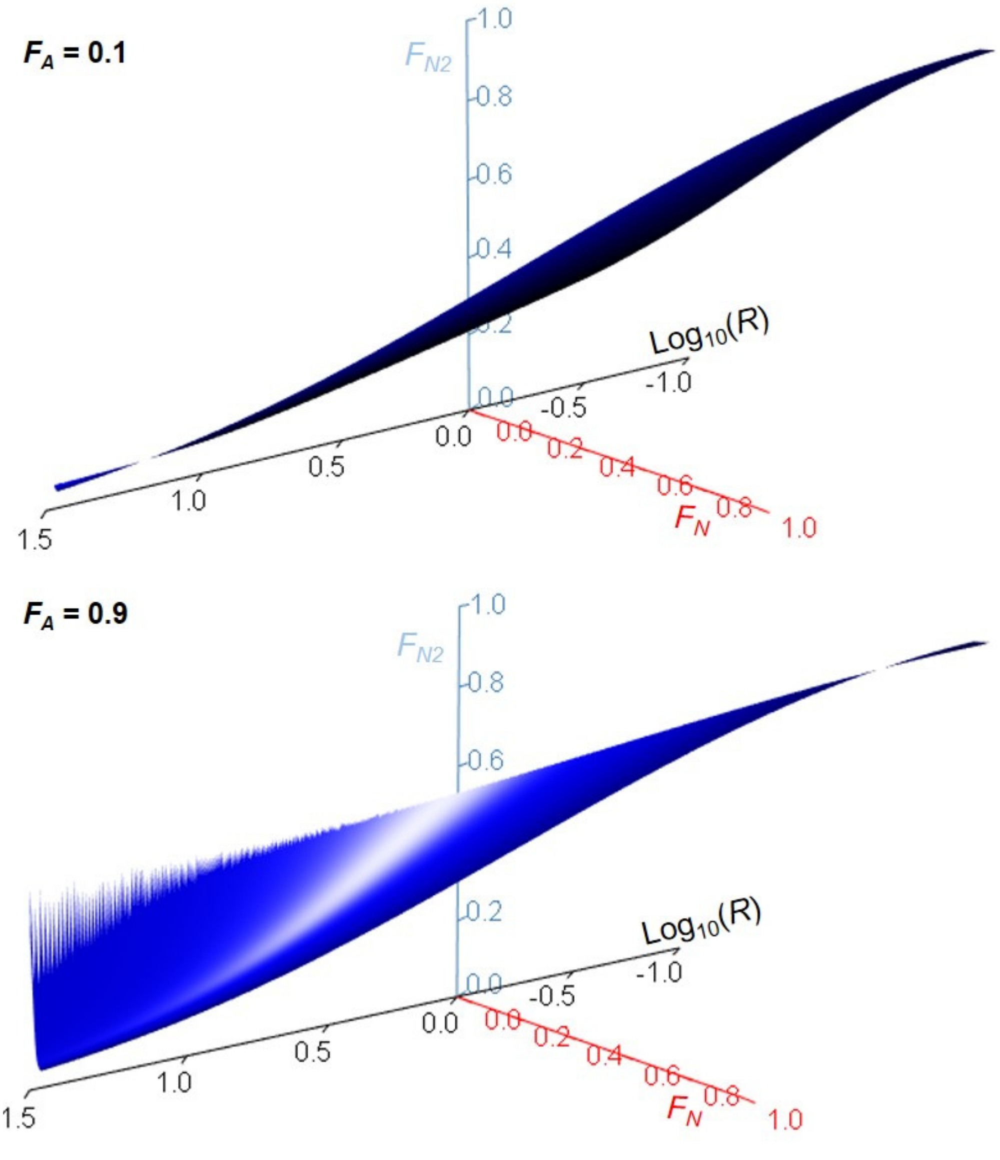

Supplementary Table 1. Site locations for the 12 rivers sampled and their sediment characteristics in rank order of gravel content (%).

			Grainsize (%)[a]			Organic C	N	C: N
River	Latitude	Longitude	gravel	sand	mud	(μmol g^{-1}) [b]	(μmol g^{-1}) [b]	(molar ratio) [b]
Pant	52.0044	0.316916	84.4	15	0.6	155±6	22±0.8	7±0
Broadstone [d]	51.08326	0.05549	82.2	15.9	1.9	255±46	17±1.8	15±1.1
Wylye	51.14248	-2.20331	74.5	23.6	1.9	555±26	49±10.7	12±1.9
Rib	51.83917	-0.02936	67.7	31.6	0.7	282±23	35±3.1	8±0.1
Lambourn*	51.44089	-1.38661	66.1	32.9	1	222±23	21±0.4	11±1
Darent*	51.35043	0.188336	53.1	44	2.8	801±59	61±6.3	13±0.4
Marden*	51.31829	-1.86	39.2	59.7	1	293±23	32±3	9±0.2
Stour (1)[c]	51.15604	0.828219	26.0	73	1	151±16	13±1.3	11±0.1
Stour (2)[c]	51.22574	0.957806	16.4	82.5	1.1	96±10	7±0.7	13±0.6
Nadder	51.04385	-2.11182	9.9	87.4	2.7	287±114	15±6.5	22±4.3
Hammer*	51.14607	0.610196	0.0	98.9	1.1	66±2	6±0.4	12±1.1
Medway	51.26798	0.518439	0.0	97.4	2.6	244±79	14±2.4	17±2.6

*The first four rivers chosen for sediment collection.

[a] Data are mean values (*n*=3).

[b] Organic C, N and C: N data are mean values ± 1 standard error (*n* = 3).

[c] The rivers Stour (1) and Stour (2) are on the chalk, with predominantly gravel-dominated riverbeds but the sediments collected from sand dominated stretches.

[d] The River Broadstone is an acidic river on a sand-based geology.

Supplementary Table 2. Contrast tables for the effects of both ATU (all 12 rivers) and addition of $^{14}NO_2^-$ (first 4 rivers) on the rates of both $^{29}N_2$ and $^{30}N_2$ production during the first 10h of oxic incubations with ^{15}N-NH_4^+. Mixed-effects models were fitted using the lme4 package in R[4] and contrasts, parameter (marginal mean) estimates and standard errors derived using emtrends with Kenwood-Roger degrees of freedom and Tukey correction where appropriate.

a, production of $^{29}N_2$ over time

Treatment (code)	Obs. (Rivers)	Parameter $^{29}N_2$ (nmol g^{-1} h^{-1})	Contrast	Estimate (s.e.)	*t*	*P*
$^{15}NH_4^+$ (2)	400 (4)	0.9997				
$^{15}NH_4^+$ + ATU (1)	400 (4)	0.0875	1 - 2	-0.9122 (0.1469)	6.211	<0.001
$^{15}NH_4^+$ + $^{14}NO_2^-$ (4)	400 (4)	1.1392	4 – 2	0.1395 (0.0966)	1.445	0.473
$^{15}NH_4^+$ + $^{14}NO_2^-$ + ATU (3)	400 (4)	0.0910	4 – 3	-1.0482 (0.1469)	7.137	<0.001
$^{15}NH_4^+$ (2)	760 (12)	0.742				
$^{15}NH_4^+$ + ATU (1)	760 (12)	0.139	1 - 2	-0.603 (0.051)	11.882	<0.001

b, production of $^{30}N_2$ over time

Treatment (code)	Obs. (Rivers)	Parameter $^{30}N_2$ (nmol g^{-1} h^{-1})	Contrast	Estimate (s.e.)	*t*	*P*
$^{15}NH_4^+$ (2)	400 (4)	0.5792				
$^{15}NH_4^+$ + ATU (1)	400 (4)	0.0026	1 - 2	-0.5766 (0.0810)	7.117	<0.001
$^{15}NH_4^+$ + $^{14}NO_2^-$ (4)	400 (4)	0.5386	4 – 2	0.0406 (0.0533)	0.761	0.872
$^{15}NH_4^+$ + $^{14}NO_2^-$ + ATU (3)	400 (4)	-0.0133	4 - 3	-0.5518 (0.0810)	6.812	<0.001
$^{15}NH_4^+$ (2)	760 (12)	0.4649				
$^{15}NH_4^+$ + ATU (1)	760 (12)	0.0484	1 - 2	-0.416 (0.029)	14.382	<0.001

Supplementary Table 3. a, Overall mean values, confidence intervals and median values for the fraction of ^{15}N labelling in the porewater NO_2^- and NO_x^- pools (F_N) and ammonia pool (F_A) for treatments 2 and 4 in the first 4 set of rivers and just treatment 2 in the second set of 12 rivers. **b,** Overall predicted *R* values as for Table 2 in the main text but for F_N as either NO_2^- or NO_x^- where appropriate. Mixed-effects models were fitted using "lme4" in R[4] and 95% confidence intervals derived using the emmeans package. Medians values were simply calculated using all observations in each treatment. Note that to estimate the means in both **a,** and **b,** incubation time was fitted as a random effect for the first set of 4 rivers but as a fixed effect for the 12 rivers.

a,

Code,	Rivers	F_N NO_2^-			F_N NO_x^-			F_A		
Treatment		Mean	95% CI	Median	Mean	95% CI	Median	Mean	95% CI	Median
**2,** $^{15}NH_4^+$	4	0.323	0.270-0.377	0.278	0.415	0.362-0.468	0.328	0.571	0.518-0.624	0.613
**4,** $^{15}NH_4^+ + ^{14}NO_2^-$	4	0.267	0.214-0.320	0.138	0.358	0.305-0.411	0.208	0.515	0.462-0.568	0.637
**2,** $^{15}NH_4^+$	12	0.160	0.128-0.192	0.111	0.250	0.218-0.282	0.187	0.448	0.416-0.480	0.469

b,

Code, Treatment	Rivers (replicates)	Process	F_N	*R* ($^{29}N_2$ / $^{30}N_2$) *Predicted*	Lower 95% C.I.	Upper 95% C.I.
**2,** $^{15}NH_4^+$	4 (5)	Denitrification	NO_2^-	7.81 (1.36)	5.1	10.5
**2,** $^{15}NH_4^+$	4 (5)		NO_x^-	7.78 (1.36)	5.1	10.5
**4,** $^{15}NH_4^+ + ^{14}NO_2$	4 (5)		NO_2^-	19.60 (1.35)	16.9	22.3
**4,** $^{15}NH_4^+ + ^{14}NO_2$	4 (5)		NO_x^-	19.54 (1.35)	16.9	22.2
**2,** $^{15}NH_4^+$	12 (5)	Denitrification	NO_2^-	29.4 (2.28)	24.8	33.9
**2,** $^{15}NH_4^+$	12 (5)	Denitrification	NO_x^-	22.2 (2.28)	17.6	26.7
**2,** $^{15}NH_4^+$	12 (5)	Anammox	NO_2^-	19.3 (2.28)	14.8	23.9
**2,** $^{15}NH_4^+$	12 (5)	Cryptic	NO_2^-	9.3 (2.28)	4.76	13.8

Supplementary References

1 Risgaard-Petersen, N., Nielsen, L. P., Rysgaard, S., Dalsgaard, T. & Meyer, R. L. Application of the isotope pairing technique in sediments where anammox and denitrification coexist. *Limnol. Oceanogr. Methods* **1**, 63 - 73 (2003).

2 Song, G. D., Liu, S. M., Kuypers, M. M. M. & Lavik, G. Application of the isotope pairing technique in sediments where anammox, denitrification, and dissimilatory nitrate reduction to ammonium coexist. *Limnol. Oceanogr. Meth.* **14**, 801-815 (2016).

3 Lansdown, K. *et al.* Importance and controls of anaerobic ammonium oxidation influenced by riverbed geology. *Nature Geosci* **9**, 357-360 (2016).

4 R Development Core Team. R: A language and environment for statistical computing. (R Foundation for Statistical Computing,Vienna, Austria., 2014).
