# Supplementary material for: Coupled nitrification and N2 gas production as a cryptic process in oxic riverbeds
Source: Nat Commun. 2021 Feb 22;12:1217. doi: 10.1038/s41467-021-21400-3 (PMC7900231; doi:10.1038/s41467-021-21400-3)
Supplement: Supplementary file 4 — Description of Additional Supplementary Files [file 41467_2021_21400_MOESM4_ESM.pdf]

Description of Additional Supplementary Files

Title: Supplementary Data 1

Description: All original and processed data. Supporting data for the 4 river study and 12 river study, each provided on two separate tabs each as either original data or after truncating and processing as described in the “Data description” tab.
